# Long-Term Outcomes of PRESBYOND Laser Correction for Presbyopia: A Retrospective Clinical Analysis

**DOI:** 10.3390/jcm15155850

**Published:** 2026-07-27

**Authors:** Ewelina Trojacka, Justyna Izdebska, Joanna Przybek-Skrzypecka, Janusz Skrzypecki, Jacek P. Szaflik

**Affiliations:** 1 Center of Ocular Microsurgery, Clinic of Prof. Jerzy Szaflik in Warsaw, 00-215 Warszawa, Poland; 2 SPKSO Ophthalmic University Hospital in Warsaw, 03-709 Warszawa, Poland; 3 Department of Ophthalmology, Medical University of Warsaw, 02-091 Warszawa, Poland; 4 Department of Experimental Physiology and Pathophysiology, Medical University of Warsaw, 02-091 Warszawa, Poland

**Keywords:** presbyopia, presbyond, laser blended vision, spectacle independence

## Abstract

**Purpose:** To evaluate the long-term outcomes of Presbyond Laser Blended Vision (LBV) in a cohort of presbyopic patients. **Methods:** This retrospective study included 100 patients (200 eyes) aged 40–69 years (mean 49.58 ± 5.76 years) who underwent Presbyond LBV between 2020 and 2024. Patients were classified as myopic (*n* = 53), hyperopic (*n* = 42), emmetropic (*n* = 3), or anisometropic (*n* = 2). All patients completed a minimum follow-up of 15 months (range, 15–59 months). Outcomes included binocular distance and near visual acuity, refractive stability, enhancement rates, patient satisfaction, visual discomfort, and spectacle independence. Owing to the very small number of emmetropic and anisometropic patients, these subgroups were evaluated descriptively only. **Results:** Between 1 month and the final follow-up (≥15 months), mean changes in spherical equivalent were small in both dominant and nondominant eyes in the myopic (−0.16 ± 0.41 D and −0.19 ± 0.60 D, respectively) and hyperopic (+0.21 ± 0.69 D and +0.18 ± 0.85 D, respectively) groups, indicating stable long-term refractive outcomes. At the final follow-up, binocular UDVA of ≤0.08 logMAR was achieved by 88.7% of myopic and 85.7% of hyperopic patients, while binocular UNVA of ≤0.30 logMAR was achieved in 99% of the overall cohort. Loss of 1–2 lines of corrected distance visual acuity occurred in 20% of patients, whereas 39.6% of myopic patients gained at least one CDVA line. Loss of near visual acuity was observed in only one hyperopic patient. Overall, 72% of patients reported satisfaction and most remained spectacle-independent. However, enhancement was required in 21% of patients, particularly among hyperopic individuals. **Conclusions:** Presbyond LBV provides durable long-term visual and refractive outcomes, excellent binocular distance and near visual acuity, and a high degree of spectacle independence in appropriately selected presbyopic patients. However, outcomes differed according to baseline refractive status. Hyperopic patients demonstrated higher enhancement rates, more frequent postoperative visual symptoms and lower subjective satisfaction despite generally favorable objective visual outcomes. These findings emphasize the importance of careful patient selection, thorough preoperative counselling and realistic expectation management, particularly in hyperopic patients. This study represents the longest-term evaluation of Presbyond LBV reported to date.

## 1. Introduction

Presbyopia is a progressive age-related loss of near vision caused by a decline in accommodative amplitude [1].

Accommodation decreases from approximately 13 D in adolescence to 6 D around the age of 40 and to about 1 D by the age of 60, progressing according to Donders’ curve at an average rate of 0.25 D per year [1].

Currently, presbyopia affects nearly 24% of the global population, with estimates predicting 2.1 billion affected individuals by 2030 due to increasing life expectancy [2]. Uncorrected presbyopia significantly reduces quality of life and may lead to decreased productivity, headaches, eye strain and tearing [3,4].

Although its exact etiology remains unclear, the most widely accepted explanation is Helmholtz’s theory, which attributes presbyopia to progressive lens stiffening caused by age-related biochemical changes [5,6]. Alternative concepts, including Schachar’s theory and modern dynamic models, additionally emphasize the role of changes within the ciliary body, choroid and vitreous body [7,8,9,10,11,12,13,14].

In response to the growing demand for spectacle independence, multiple surgical strategies have been developed, including monovision, multifocality and extended depth of focus (EDOF) approaches [15]. However, conventional monovision and multifocal techniques may reduce stereoacuity and contrast sensitivity [16,17,18,19,20,21].

Presbyond (Laser Blended Vision, Carl Zeiss Meditec, London, United Kingdom), introduced by Dan Reinstein, is an advanced corneal EDOF technique combining non-linear aspheric ablation with micro-monovision using femtoLASIK and MEL 80/90 excimer laser platforms [22,23,24,25]. The method increases depth of focus by inducing controlled spherical aberrations and targets emmetropia in the dominant eye with mild myopia (approximately −1.50 D) in the non-dominant eye [22,23,24,25]. Unlike traditional monovision, Presbyond creates a smooth binocular “blend zone” that enables functional vision at near, intermediate and distance ranges while preserving good distance visual acuity, contrast sensitivity and stereopsis [26,27,28,29,30,31].

### Aim of the Study

While the effectiveness of the Presbyond method is well documented in the international literature, published clinical outcomes are predominantly limited to follow-up periods of up to 12 months after surgery. Moreover, clinical results may vary depending on the preoperative refractive status of the patient. The aim of the present study is to analyze the clinical outcomes of the Presbyond procedure in a cohort of 100 patients with an extended follow-up period of 15 months or longer. The study evaluates visual acuity, refractive stability (long-term refractive outcomes) and patient satisfaction in a clinical population, including individuals with myopia, hyperopia, emmetropia and anisometropia.

## 2. Materials and Methods

### 2.1. Ethical Approval

This retrospective observational study was conducted in accordance with the principles of the Declaration of Helsinki. The study protocol was reviewed by the Bioethics Committee of the Regional Medical Chamber in Warsaw, Poland. The Committee issued Opinion No. KB/1816/26 (reference number KB.820.011/2026.0001) and determined that due to the retrospective nature of the study and the use of anonymized clinical data, formal ethical approval and informed consent were not required.

All patient data were fully anonymized prior to analysis to ensure confidentiality and compliance with data protection regulations.

### 2.2. Study Design and Objectives

This retrospective clinical study was designed to evaluate the long-term outcomes of the Presbyond LBV (Laser Blended Vision, Carl Zeiss Meditec, London, United Kingdom) procedure based on the analysis of existing medical records. The primary objective was to assess the efficacy, safety and refractive stability of the correction over a follow-up period of at least 15 months after surgery. The study focused on the performance of the Presbyond technique in a population of presbyopic patients with preoperative myopia, hyperopia, emmetropia and anisometropia.

### 2.3. Patient Population

The study cohort consisted of 100 patients (200 eyes) who underwent Presbyond LBV surgery at the Centre of Eye Microsurgery Laser in Warsaw, Poland. Patients were identified retrospectively based on the availability of complete clinical documentation, including preoperative qualification and postoperative follow-up visits.

To enable a detailed analysis, patients were categorized according to their preoperative spherical equivalent:

- Myopic group: spherical equivalent from −0.50 D to −11.00 D;
- Hyperopic group: spherical equivalent from +0.50 D to +6.00 D;
- Emmetropic group: spherical equivalent within ±0.50 D;
- Anisometropic group: different refractive status between fellow eyes, defined as an interocular difference in spherical refractive error of ≥2.50 D.

All included patients were 40 years of age or older and presented with clinically significant presbyopia requiring optical correction for near vision.

### 2.4. Preoperative Screening and Inclusion Criteria

Preoperative qualification data were obtained from medical records of visits preceding surgery. Stringent eligibility criteria had been applied at the time of patient selection for the procedure and were used as inclusion criteria for the present analysis:

- Refractive stability: no significant change in refractive error for at least 12 months prior to surgery;
- Visual acuity: corrected distance visual acuity (CDVA) of 0.15 logMAR or better in each eye;
- Monovision tolerance: successful adaptation confirmed by a preoperative cross-blur tolerance test, demonstrating the patient’s ability to tolerate induced anisometropia between 0.75 D and 1.50 D;
- Corneal status: normal corneal topography and tomography (Galilei G4 and MS-39), excluding subclinical ectasia or irregular astigmatism.

Patients with significant crystalline lens opacities (cataract), advanced dry eye disease, glaucoma, retinal pathology, or any other coexisting ocular disorders were excluded from the analysis.

### 2.5. Surgical Technique

All procedures were performed using a Femto-LASIK approach. Corneal flap creation was achieved with the VISUMax 500/800 femtosecond laser (Carl Zeiss Meditec, Jena, Germany), followed by stromal ablation using the MEL 80/90 excimer laser (Carl Zeiss Meditec, Jena, Germany).

The ablation profile was calculated using CRS Master software 2.3.0 and included:

- Personalized non-linear aspheric ablation adjusted to the patient’s preoperative spherical aberration profile
- Bilateral induction of controlled spherical aberration aimed at increasing the depth of focus in both the dominant (distance) and nondominant (near) eye
- A micro-monovision strategy targeting emmetropia in the dominant eye and residual myopia between −0.75 D and −1.50 D in the nondominant eye, resulting in the creation of a functional blend zone

### 2.6. Follow-Up Protocol and Outcome Measures

Clinical data were collected retrospectively from documented postoperative visits, including the routine follow-up at approximately 1 month after surgery and the last available examination performed at least 15 months postoperatively. Intermediate follow-up visits between these time points were not included in the analysis, as the primary aim of the study was to evaluate long-term outcomes. Short-term postoperative results have already been extensively reported in the literature [28,29,30,31,32,33,34,35,36].

Patients who underwent enhancement procedures remained in the study cohort. These patients were evaluated at the same predefined final follow-up visit, which was performed at least 15 months after the enhancement procedure. Consequently, the final analyses represent the long-term clinical outcomes of the complete treatment pathway, including both the primary Presbyond procedure and any clinically indicated enhancement.

The following outcome measures were analyzed:

- Efficacy: binocular uncorrected distance visual acuity (UDVA) and binocular uncorrected near visual acuity (UNVA)
- Refractive stability: spherical equivalent (SE) and its evolution over time
- Safety: changes in binocular distance and near visual acuity, including loss of lines of corrected visual acuity
- Patient-reported outcomes: spectacle independence, subjective visual comfort, and overall satisfaction

At the final follow-up visit, patients completed a standardized questionnaire consisting of closed-ended questions based on a three-point Likert scale. Satisfaction with the surgical outcome was classified as satisfied, moderately satisfied, or dissatisfied. Spectacle use was classified as yes, sometimes, or no.

### 2.7. Statistical Analysis

Continuous variables are presented as mean ± standard deviation (SD) and categorical variables as counts and percentages. All statistical tests were two-sided, and a *p*-value < 0.05 was considered statistically significant.

For refractive outcomes, spherical equivalent (SE) was analyzed on an eye-level basis using generalized estimating equations (GEE) with a Gaussian distribution, robust standard errors, patient-level clustering and an exchangeable working correlation structure to account for inter-eye dependency. The model included time point, eye dominance and their interaction, enabling separate estimation of postoperative changes in dominant and nondominant eyes. Two planned comparisons were performed: preoperative versus 1 month postoperative (early refractive effect) and 1 month versus ≥15 months postoperative (long-term refractive stability).

Age-stratified refractive stability was assessed using the change in spherical equivalent (ΔSE), calculated as the difference between the final follow-up (≥15 months) and the 1-month postoperative examination. Patients were categorized into the following predefined age groups: 40–45, 46–50, 51–55, 56–60, and >60 years. Within each age subgroup, ΔSE was analyzed using GEE models clustered by patient to account for inter-eye correlation. Groups with insufficient sample size or no variability were reported descriptively only.

Visual acuity outcomes, including binocular preoperative best-corrected distance visual acuity (BCDVA), postoperative uncorrected distance visual acuity (UDVA), preoperative best-corrected near visual acuity (BCNVA) and postoperative uncorrected near visual acuity (UNVA), were analyzed on a per-patient basis. Efficacy was assessed by comparing preoperative BCDVA with postoperative UDVA at the final follow-up. Stability was assessed by comparing outcomes obtained at 1 month and ≥ 15 months postoperatively. The same approach was applied to near visual acuity.

Categorical variables, including changes in corrected distance visual acuity (CDVA) lines, spectacle correction requirements, enhancement rates, postoperative visual discomfort and patient satisfaction, were analyzed descriptively and reported as proportions. 

Detailed statistical analyses supporting the reported findings are available in Supplementary Files S1 and S2.

During the preparation of this manuscript, the author(s) used ChatGPT (OpenAI, GPT-5.5) to assist with statistical interpretation, data visualization and figure preparation.

## 3. Results

### 3.1. Study Population

A total of 100 patients (200 eyes) undergoing Presbyond laser vision correction were included in the analysis. 53 patients were initially myopic, 42 hyperopic, 2 anisometropic and 3 emmetropic. Patient age ranged from 40 to 69 years, with a mean age of 49.58 ± 5.755 years. The minimum baseline spherical equivalent was −10.63 D, and the maximum was +5.00 D.

The minimum follow-up duration was 15 months, and the maximum was 59 months. The mean follow-up duration was 31.06 ± 11.889 months. All patients completed long-term follow-up of at least 15 months.

Visual acuity outcomes (distance and near) were analyzed per patient (binocular values), while refractive outcomes were analyzed per eye with separate evaluation of dominant and non-dominant eyes. 

Baseline characteristics of the study population by refractive group are summarized in Table 1.

### 3.2. Myopic Group

In the myopic subgroup (*n* = 53), refractive correction resulted in a marked and statistically significant improvement in both dominant and non-dominant eyes between baseline and 1 month postoperatively (*p* < 0.001). Although small but statistically significant changes were observed between 1 month and ≥15 months (dominant eye *p* = 0.003; non-dominant eye *p* = 0.020), these did not represent clinically meaningful regression and overall refractive stability was maintained over time. The detailed results are presented in Table 2.

Table 2, Table 3 and Table 4 summarize refractive outcomes (SE), binocular visual acuity outcomes and efficacy, and safety results.

Binocular distance visual acuity remained stable throughout follow-up. At ≥15 months, postoperative UDVA did not differ significantly from preoperative BCDVA (*p* = 0.08), and no significant change was observed between the 1-month and final examinations (*p* = 0.136). At the last follow-up visit, 88.7% of patients achieved binocular UDVA of 0.08 logMAR or better.

Binocular near visual acuity also remained stable across all follow-up visits. No statistically significant differences were observed either between baseline and ≥15 months or between analyzed postoperative visits, and no loss or gain of NVA lines was recorded. The distribution of visual acuity is presented in Table 3.

With respect to safety, changes in CDVA showed that 39.6% of patients gained at least one line and 41.5% remained unchanged, whereas 18.9% lost at least one line, including 11.3% who lost two or more lines.

Visual comfort was assessed at the final follow-up visit (≥15 months postoperatively). Postoperative visual discomfort was relatively common and was reported by 24.5% of patients for distance vision, 20.8% for near vision, and 9.4% for intermediate vision. Spectacle correction at the final follow-up was required by 9.4% of patients for distance vision, 7.5% for near vision, and 5.7% for intermediate vision. Overall, visual discomfort was reported by 27 of 53 patients (50.9%).

Enhancement was performed in 9 patients, with the interval between the primary procedure and the secondary intervention ranging from 5 to 14 months. Despite the additional surgery, visual discomfort persisted in 4 of the 9 reoperated patients (44.4%). At the final follow-up visit, 8 of the 9 reoperated patients reported dissatisfaction with the surgical outcome.

Despite the relatively frequent reporting of visual disturbances, overall patient satisfaction remained high, with 75.5% of patients reporting satisfaction with the surgical outcome at the final follow-up visit. 

A detailed summary of the efficacy and safety outcomes is provided in Table 5.

**Figure 1:**
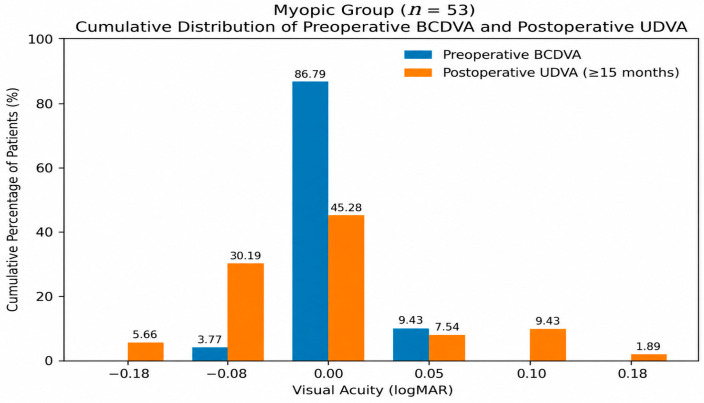
Cumulative histogram showing efficacy of myopia treatment: postoperative UDVA and preoperative BCDVA.

**Figure 2:**
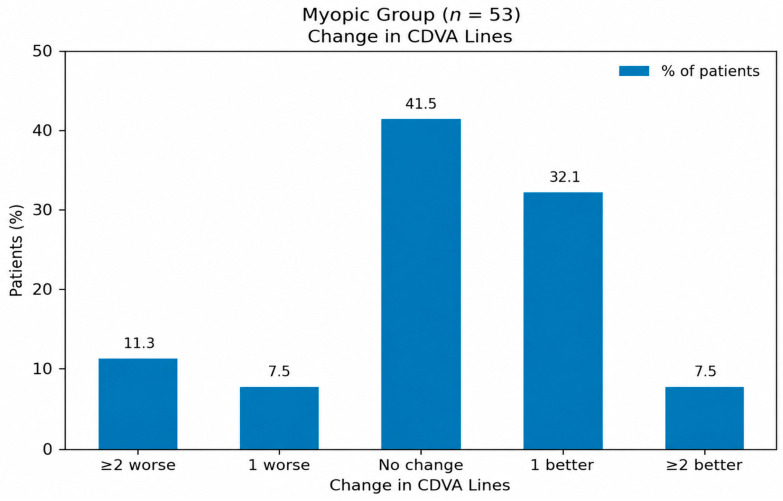
Histogram illustrating the change in CDVA values for the whole myopia subgroup.

**Figure 3:**
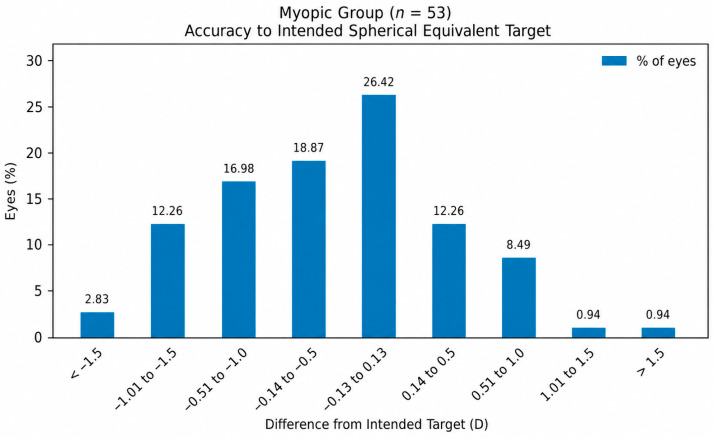
Histogram presenting the accuracy to the intended spherical equivalent refraction for the myopia sample.

**Figure 4:**
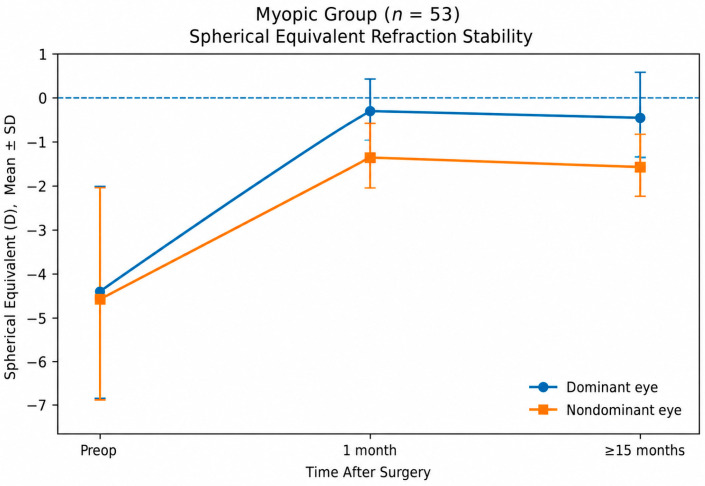
Spherical equivalent refraction stability of myopic patients at each time point after surgery.

The refractive and visual outcomes for the myopic subgroup are presented in Figure 1, Figure 2, Figure 3 and Figure 4.

### 3.3. Hyperopic Group

In the hyperopic subgroup (*n* = 42), refractive correction resulted in a significant change in spherical equivalent between baseline and 1 month postoperatively in both dominant and non-dominant eyes (*p* < 0.001). Thereafter, refractive outcomes remained stable, with only small and statistically non-significant shifts observed between 1 month and ≥15 months (dominant eye *p* = 0.051; non-dominant eye *p* = 0.178). The detailed results are presented in Table 2.

Binocular distance visual acuity showed an improvement over time. UDVA improved significantly between the 1-month and ≥15-month examinations (*p* = 0.002), and final UDVA did not differ significantly from preoperative BCDVA (*p* = 0.405). At the final follow-up visit, 85.7% of patients achieved binocular UDVA of 0.08 logMAR or better.

Binocular near visual acuity remained stable throughout the observation period. No statistically significant differences were found between baseline and ≥15 months (*p* = 0.323) or between the 1-month and final examinations (*p* = 1.000). A loss of two lines of NVA was observed in one patient (2.38%). 

The distribution of visual acuity is presented in Table 3.

With regard to safety, CDVA outcomes showed that 23.8% of patients gained at least one line and 57.1% remained unchanged, whereas 19.05% lost at least one line, including 11.9% who lost two or more lines. 

Visual comfort was assessed at the final follow-up visit (≥15 months postoperatively), representing the late postoperative period. Postoperative visual discomfort was reported by 45.24% of patients for distance vision, 28.57% for near vision and 11.9% for intermediate vision. Overall, visual discomfort was reported by 26 of 42 patients (60.9%). Spectacle correction at the final follow-up was required by 9.5% of patients for distance vision, 7.1% for near vision and 2.4% for intermediate vision.

Enhancement surgery was required in 12 patients (28.6%), with the interval between the primary procedure and the secondary intervention ranging from 1.5 to 22 months. Despite the additional surgery, visual discomfort persisted in 3 of the 12 reoperated patients (25.0%). At the final follow-up visit, all reoperated patients (12/12) reported dissatisfaction with the surgical outcome.

Despite the relatively frequent reporting of visual discomfort, overall patient satisfaction remained high, with 64.3% of patients reporting satisfaction with the surgical outcome. 

A detailed summary of the efficacy and safety outcomes is provided in Table 5.

**Figure 5:**
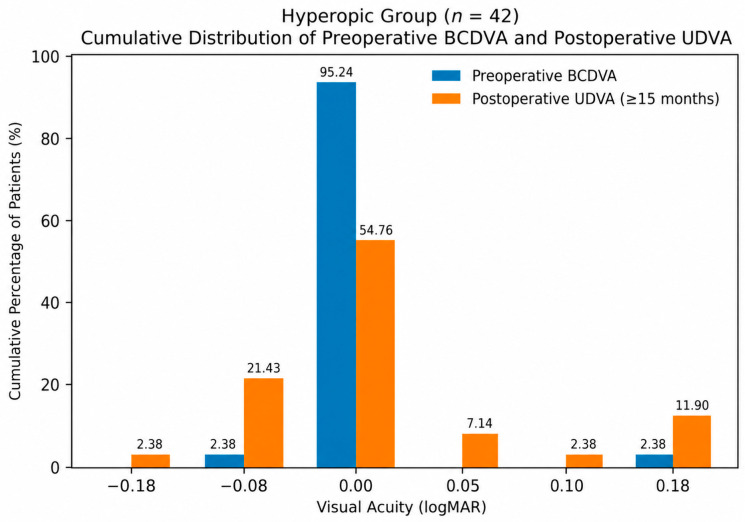
Cumulative histogram showing efficacy of hyperopia treatment: postoperative UDVA and preoperative BCDVA.

**Figure 6:**
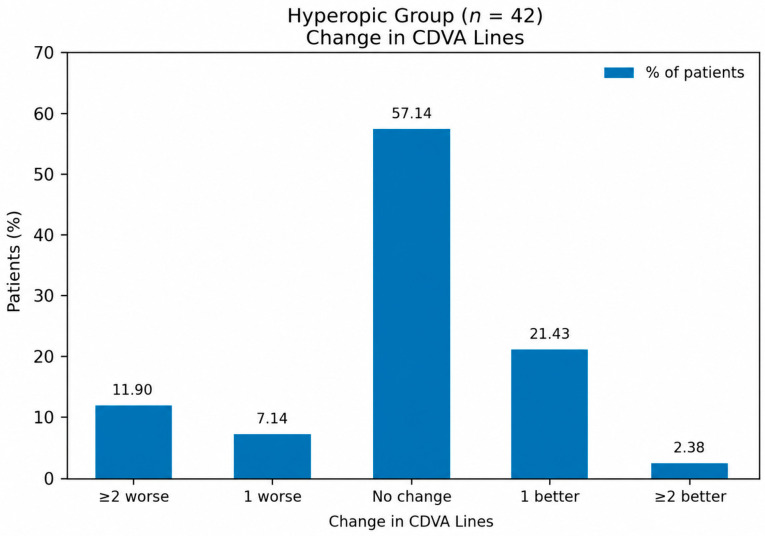
Histogram presenting the change in CDVA values for the whole hyperopia subgroup.

**Figure 7:**
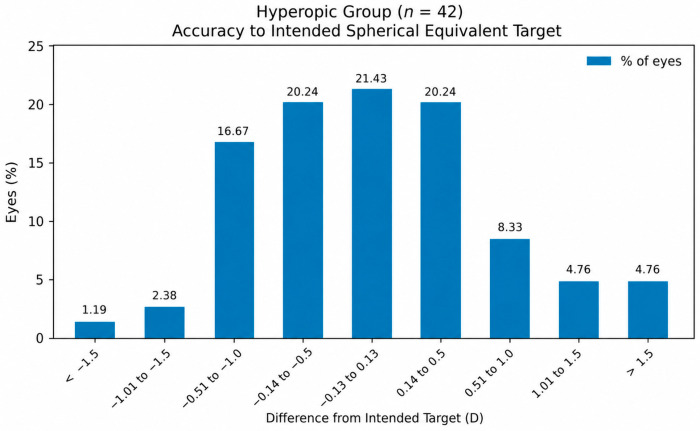
Histogram depicting the accuracy to the intended spherical equivalent refraction for the hyperopia sample.

**Figure 8:**
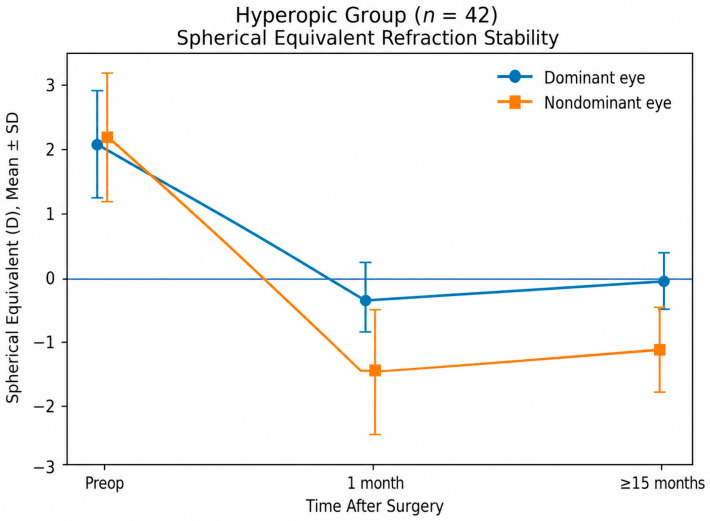
Spherical equivalent refraction stability of hyperopic patients at each time point.

The refractive and visual outcomes for the hyperopic subgroup are presented in Figure 5, Figure 6, Figure 7 and Figure 8.

### 3.4. Anisometropic and Emmetropic Group

Three emmetropic and two anisometropic patients were included in the study. Owing to the very limited sample sizes, no inferential statistical analysis was performed for these subgroups, and the findings should be considered descriptive only.

In the emmetropic subgroup, two of the three patients achieved a final binocular UDVA of ≤0.08 logMAR, whereas one patient had a final UDVA > 0.08 logMAR. Regarding safety, one patient lost two or more CDVA lines, one patient lost one CDVA line, and one patient showed no change in CDVA. No patient gained CDVA lines. No enhancement procedures were required. None of the patients required spectacles for distance or intermediate vision at the final follow-up, while one patient reported spectacle use for near vision. All three patients reported satisfaction with the surgical outcome.

In the anisometropic subgroup, both patients achieved a final binocular UDVA of ≤0.08 logMAR. CDVA remained unchanged throughout follow-up, with no loss or gain of CDVA lines observed. Neither enhancement procedures nor postoperative spectacle correction was required, and both patients reported satisfaction with the surgical outcome.

Because of the extremely small sample sizes, these observations should not be interpreted as evidence of the effectiveness or safety of Presbyond LBV in emmetropic or anisometropic patients. Larger studies are required to evaluate outcomes in these specific refractive groups.

### 3.5. Age-Stratified Analysis of Refractive Stability

Age-stratified analysis of refractive stability was performed by evaluating the change in spherical equivalent (ΔSE), calculated as the difference between the final follow-up (≥15 months) and the 1-month postoperative examination. For each age subgroup, the mean ΔSE was compared against zero using a one-sample t-test. Patients were categorized into the following predefined age groups: 40–45, 46–50, 51–55, 56–60 and >60 years.

In the myopic group, a statistically significant mean change in spherical equivalent was observed only in the 40–45 years age category (mean ΔSE = −0.268 ± 0.359 D, *p* < 0.001). In the remaining age groups (46–50, 51–55 and 56–60 years), the mean change did not differ significantly from zero (*p* = 0.872, *p* = 0.077 and *p* = 0.070, respectively).

In the hyperopic group, statistically significant mean changes in spherical equivalent were observed in patients aged 46–50 years (*p* = 0.032) and 51–55 years (*p* = 0.015). In contrast, no significant changes were detected in the 40–45, 56–60 and >60 years age categories (*p* = 0.549, *p* = 0.290 and *p* = 0.453, respectively).

In the emmetropic group, the largest refractive change was observed in the oldest patient, belonging to the 56–60 years age category; however, interpretation is limited by the extremely small sample size. In the anisometropic group, both patients belonged to the same 46–50 years age category; therefore, age-stratified analysis was not feasible.

Overall, no consistent age-related pattern of refractive change was observed. Although greater variability of ΔSE was noted in some older subgroups, particularly among patients aged ≥ 56 years, these findings should be interpreted with considerable caution. Several predefined age categories included only a small number of patients, whereas others were not represented, resulting in limited statistical power and wide variability. Consequently, these observations should be regarded as exploratory and require confirmation in larger cohorts with a more balanced age distribution.

**Figure 9:**
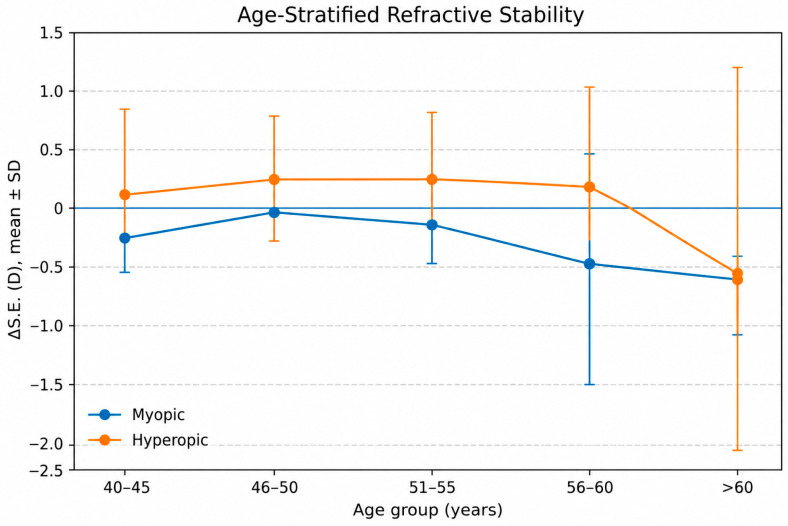
Age-stratified refractive stability expressed as the change in spherical equivalent (ΔS.E.) between the 1-month postoperative visit and the final follow-up (≥15 months) across myopic and hyperopic refractive groups. Values are presented as mean ± SD.

Detailed age-stratified results for refractive stability are presented in Table 5 and Figure 9.

When patient-reported outcomes were analyzed according to age categories and refractive groups, differences were observed in patient satisfaction, enhancement rates, and the need for spectacle correction.

In the myopic group, patient satisfaction remained relatively high across most age categories. Satisfaction ranged from 70.0% in patients aged 51–55 years to 84.2% in the 46–50 age group. A decrease in satisfaction was observed in the 56–60 age group (50%), which was accompanied by higher enhancement rates (50%) and a higher proportion of patients requiring spectacle correction postoperatively (50%). In younger age groups, the proportion of patients requiring enhancement remained relatively low, ranging from 10.5% to 20%.

In contrast, outcomes in the hyperopic group were more heterogeneous across age categories. Satisfaction increased from 40% in the 40–45 age group to 82.4% in patients aged 51–55 years, representing the highest level of satisfaction in this group. However, lower satisfaction was observed in the youngest and oldest presbyopic patients, with no satisfaction reported among patients aged > 60 years. The frequency of enhancement procedures was also relatively high in these groups, reaching 60.0% in patients aged 40–45 years and 100% in those aged > 60 years, with a substantial proportion of older patients requiring postoperative spectacle correction (66.7%).

In the emmetropic group, three patients were included, each belonging to a different age category (40–45, 46–50 and 56–60 years). All patients reported satisfaction with the surgical outcome and no enhancement procedures were required. Spectacle correction after surgery was reported in only one patient. Because of the very small number of patients in this group, the results should be interpreted cautiously.

Similarly, the anisometropic group included only two patients, both belonging to the 46–50 age category. Both patients reported satisfaction with the surgical outcome and no enhancements or postoperative spectacle dependence were observed. Due to the extremely small sample size, these findings should be considered descriptive only.

Given the limited sample size and lack of representation in several predefined age categories, age-stratified findings should be interpreted with caution and considered exploratory. Further studies including larger cohorts with a more balanced age distribution are warranted to confirm these observations.

Detailed age-stratified results for satisfaction, reoperation rates and postoperative spectacle dependence are presented in Table 6.

### 3.6. Enhancement Procedures

Enhancement was performed in 21 patients (21.0%), including 9 myopic patients (17.0%) and 12 hyperopic patients (28.6%). The mean time from the primary Presbyond procedure to enhancement was 8.3 ± 2.9 months in the myopic group and 10.7 ± 6.5 months in the hyperopic group.

All patients, including those who underwent enhancement procedure, completed a minimum follow-up of 15 months after their last surgical intervention. Therefore, the final visual acuity and refractive analyses represent the ultimate long-term postoperative status of the entire cohort.

The indications for enhancement included postoperative refractive outcomes deviating from the intended target, refractive regression and clinically significant visual discomfort interfering with daily activities. Among patients reporting visual discomfort, the most common complaint in the myopic group was impaired distance vision (4 patients), followed by near vision (2 patients), intermediate vision (2 patients) and symptoms affecting more than one viewing distance (1 patient). In the hyperopic group, distance vision was also the most frequently affected (5 patients), followed by near vision (4 patients) and more than one viewing distance (3 patients). No patient in the hyperopic group underwent enhancement because of isolated intermediate-vision complaints.

All enhancement procedures were performed by flap lift followed by additional excimer laser ablation.

At the predefined final follow-up examination, enhanced myopic patients achieved a mean binocular UDVA of 0.00 ± 0.08 logMAR and a mean binocular UNVA of 0.30 ± 0.00 logMAR. The mean spherical equivalent was −0.42 ± 0.69 D in the dominant eye and −2.34 ± 1.16 D in the nondominant eye. Comparable outcomes were observed in enhanced hyperopic patients, who achieved a mean binocular UDVA of 0.03 ± 0.07 logMAR and a mean binocular UNVA of 0.28 ± 0.09 logMAR. The mean spherical equivalent was −0.05 ± 0.46 D in the dominant eye and −1.33 ± 1.22 D in the nondominant eye.

Despite enhancement, persistent visual discomfort at the final follow-up was reported by 4 of 9 myopic patients (44.4%) and 3 of 12 hyperopic patients (25.0%).

## 4. Discussion

The present study provides the longest follow-up currently reported for Presbyond LBV, with postoperative observation ranging from 15 to 59 months. While previous studies have consistently demonstrated favorable short- and medium-term outcomes following Presbyond treatment, published follow-up has generally been limited to 6–12 months [25,29,30,31,32,33,34,35,36].

The present findings extend the available evidence by demonstrating that good binocular distance and near visual acuity, high spectacle independence and durable refractive outcomes can be maintained over substantially longer periods. Importantly, the study also provides a comprehensive assessment of enhancement procedures, postoperative visual symptoms and patient-reported satisfaction, allowing a more complete evaluation of long-term clinical performance than has been reported previously.

Our results are generally consistent with those of previous Presbyond studies. Ganesh et al., Brar et al., Russo et al., Van Heerden et al., Hernández-Lucena et al., and Fu et al. reported excellent refractive accuracy, functional binocular vision, and high patient satisfaction following Presbyond surgery [25,29,30,31,33,35,36]. However, these investigations were limited by relatively short follow-up periods and therefore could not determine whether the favorable early postoperative outcomes remained stable over time. In contrast, the present study demonstrates that the visual and refractive benefits achieved after surgery are largely maintained over follow-up extending to almost five years. Consequently, our findings complement the existing literature by providing evidence for the long-term durability of the Presbyond optical concept under routine clinical conditions.

The refractive outcomes observed in the present study confirm that Presbyond provides sustained long-term refractive correction in both myopic and hyperopic patients. As expected, statistically significant changes in spherical equivalent were observed between the preoperative examination and the first postoperative visit, reflecting the intended refractive correction. Thereafter, only small changes occurred between the 1-month and final follow-up examinations. Although some comparisons reached statistical significance, the magnitude of these changes remained clinically small and did not appear to compromise binocular visual performance or spectacle independence. This distinction between statistical significance and clinical relevance is particularly important when interpreting longitudinal refractive outcomes in ophthalmic studies.

An important methodological aspect of the present analysis is that refractive outcomes were evaluated after completion of the entire treatment pathway rather than after the primary procedure alone. Patients who required enhancement remained in the study cohort, and all completed a minimum follow-up of 15 months after their last surgical intervention. Consequently, the final visual acuity and refractive analyses represent the ultimate long-term clinical status of the entire cohort after all clinically indicated treatments had been completed. Therefore, the reported enhancement rate should not be interpreted as contradicting the favorable long-term outcomes but rather as reflecting the proportion of patients who required additional treatment to achieve their final postoperative result. In this context, the term long-term refractive outcomes refers to the final sustained refractive status after completion of treatment rather than the absence of secondary procedures.

The age-stratified analysis did not demonstrate a consistent relationship between patient age and long-term refractive outcomes. Although statistically significant refractive shifts were identified in selected age categories, these findings should be interpreted cautiously. Several age subgroups contained only a small number of patients, while some age categories were not represented at all, resulting in substantial variability and wide standard deviations. Consequently, these analyses should be regarded as exploratory and hypothesis-generating rather than providing evidence of true age-dependent differences. Nevertheless, the observed tendency toward greater refractive variability among older hyperopic patients may reflect progressive lenticular changes and warrants further investigation in larger prospective cohorts.

One of the most clinically relevant findings of the present study is the difference in postoperative course between myopic and hyperopic patients. Although both groups achieved excellent binocular visual acuity and high levels of spectacle independence, hyperopic patients experienced a more challenging postoperative course characterized by higher enhancement rates, more frequent postoperative visual symptoms and lower subjective satisfaction. Enhancement procedures were required in 28.6% of hyperopic patients compared with 17.0% of myopic patients, while postoperative visual discomfort was reported by 60.9% and 50.9% of patients, respectively. Importantly, despite undergoing enhancement, persistent visual discomfort remained present in a proportion of patients at the final follow-up, emphasizing that optimization of refractive error alone does not invariably restore complete subjective visual comfort.

Several mechanisms may explain these findings. Hyperopic laser ablation profiles are generally considered more demanding than myopic treatments because tissue removal occurs predominantly in the peripheral cornea, making outcomes more susceptible to centration errors, epithelial remodeling and corneal biomechanical changes. Furthermore, hyperopic patients are typically older than myopic patients at the time of surgery, increasing the likelihood of early crystalline lens dysfunction despite the absence of clinically significant cataract. Progressive lenticular changes, together with continuing loss of accommodation and neural adaptation, may contribute to postoperative visual symptoms, lower patient satisfaction, and the increased likelihood of requiring enhancement during long-term follow-up. These observations emphasize that refractive status, patient age, crystalline lens status and visual expectations should all be carefully considered during patient selection and preoperative counselling.

The enhancement analysis provides important insight into the long-term management of patients undergoing Presbyond LBV. Overall, enhancement was required in 21.0% of patients, with a substantially higher frequency in the hyperopic group than in the myopic group (28.6% vs. 17.0%). The mean interval between the primary procedure and enhancement was 8.3 ± 2.9 months in myopic patients and 10.7 ± 6.5 months in hyperopic patients, indicating that most secondary interventions were performed during the first postoperative year after stabilization of the initial refractive outcome.

Enhancement was performed only when objective clinical findings corresponded with clinically significant subjective symptoms. The primary indications included postoperative refractive outcomes deviating from the intended target, refractive regression and unacceptable visual discomfort interfering with daily activities. Importantly, enhancement decisions were based not only on residual refractive error but also on patient-reported functional impairment, reflecting the contemporary concept that successful presbyopia correction should be evaluated using both objective refractive outcomes and subjective visual performance.

All enhancement procedures were performed by flap lift followed by excimer laser ablation. At the final follow-up examination, enhanced patients achieved satisfactory binocular visual acuity. In the myopic group, mean binocular UDVA and UNVA were 0.00 ± 0.08 logMAR and 0.30 ± 0.00 logMAR, respectively, whereas corresponding values in the hyperopic group were 0.03 ± 0.07 logMAR and 0.28 ± 0.09 logMAR. Likewise, the final spherical equivalent remained close to the intended target in both dominant and nondominant eyes. Nevertheless, persistent postoperative visual discomfort remained present in four of nine enhanced myopic patients and three of twelve enhanced hyperopic patients at the final examination. These findings indicate that although enhancement effectively optimized refractive outcomes in most cases, improvement in objective refractive parameters does not necessarily translate into complete restoration of subjective visual comfort.

This discrepancy between objective clinical outcomes and subjective patient satisfaction represents one of the most clinically relevant observations of the present study. Conventional refractive outcome measures, including spherical equivalent and high-contrast visual acuity, provide only a partial assessment of postoperative visual quality. Patients undergoing presbyopia correction frequently report symptoms such as reduced visual comfort, difficulties during prolonged reading, impaired intermediate vision, glare, halos, reduced contrast sensitivity, or visual fatigue, despite achieving excellent Snellen or logMAR acuity. Consequently, patient-reported outcome measures should be regarded as an essential component of the evaluation of modern presbyopia surgery.

The higher frequency of postoperative visual discomfort reported by hyperopic patients deserves particular attention. Although objective visual acuity remained excellent in most cases, visual discomfort persisted in more than half of hyperopic patients, and subjective dissatisfaction remained relatively common despite enhancement. These findings suggest that patient expectations may be more difficult to satisfy in hyperopic presbyopes than in myopic patients. This observation is consistent with previous reports indicating that hyperopic laser correction is generally associated with lower refractive predictability, greater biological variability and increased sensitivity to subtle lenticular changes compared with myopic treatments.

The present findings should also be interpreted within the broader context of currently available surgical options for presbyopia correction. As summarized in a recent comprehensive review of contemporary presbyopia correction strategies, no single surgical technique is universally optimal, and treatment should be individualized according to patient age, refractive error, crystalline lens status, occupational requirements, lifestyle, and visual expectations [37].

Conventional monovision remains one of the simplest and most widely used approaches for surgical correction of presbyopia. Although it provides good functional vision in appropriately selected patients, larger degrees of anisometropia may reduce stereopsis, binocular summation and contrast sensitivity, while a proportion of patients fail to adapt to induced monovision. Presbyond differs fundamentally from conventional monovision by combining low anisometropia with controlled induction of spherical aberration, thereby increasing binocular depth of focus while attempting to preserve binocular visual quality and facilitate neural adaptation.

Other corneal laser procedures, including multifocal ablation profiles such as PresbyMAX and SUPRACOR, generate simultaneous multifocality on the corneal surface, and have demonstrated excellent near visual acuity. However, these techniques may also increase photic phenomena and reduce contrast sensitivity because incoming light is divided between multiple focal points. In contrast, Presbyond primarily relies on an extended depth-of-focus concept rather than true multifocality, aiming to preserve distance image quality while maintaining functional vision across multiple working distances. This optical concept may explain the consistently favorable binocular distance vision reported in previous studies and the present investigation.

Lens-based procedures, including refractive lens exchange with multifocal or extended depth-of-focus intraocular lenses, represent an excellent alternative for older presbyopic patients or those with early crystalline lens dysfunction. These procedures eliminate future cataract development and generally provide highly predictable refractive correction. However, they expose patients to the inherent risks of intraocular surgery, including retinal detachment, endophthalmitis, posterior capsule opacification, dysphotopsias and potential secondary interventions. Consequently, corneal procedures such as Presbyond remain particularly attractive in younger presbyopic patients with a clear crystalline lens who seek spectacle independence while preserving their natural lens.

Although direct comparisons between different presbyopia correction strategies remain difficult because of considerable heterogeneity in patient selection, outcome measures and follow-up duration, the present study demonstrates that Presbyond provides durable long-term binocular visual performance and high levels of spectacle independence over follow-up extending to 59 months. These findings further support its role as an effective corneal extended-depth-of-focus procedure within the current spectrum of surgical presbyopia correction while emphasizing that appropriate patient selection remains fundamental to achieving optimal long-term outcomes.

Overall, the present study demonstrates that Presbyond LBV provides favorable long-term functional outcomes. However, these results should be interpreted alongside the observed enhancement rate and postoperative visual discomfort. Although most patients maintained or improved corrected distance visual acuity, approximately one-fifth experienced a loss of at least one CDVA line. Furthermore, postoperative visual discomfort remained relatively common, particularly among hyperopic patients, despite generally good objective refractive outcomes. These findings emphasize that the success of presbyopia surgery should not be evaluated solely on the basis of refractive accuracy or high-contrast visual acuity but should also incorporate patient-reported visual quality and everyday visual function.

The present study also highlights the importance of careful patient selection and preoperative counselling. In particular, hyperopic patients should be informed that although excellent binocular visual acuity and high levels of spectacle independence can be achieved, they may have a greater likelihood of requiring enhancement and may experience persistent postoperative visual disturbances despite satisfactory objective refractive outcomes. Establishing realistic expectations before surgery is therefore essential for optimizing long-term patient satisfaction.

### Strengths and Limitations

The principal strength of the present study is the longest follow-up currently reported for Prebyond LBV, extending to 59 months after surgery. Unlike previous studies, which primarily focused on early postoperative outcomes, the present investigation provides comprehensive long-term evaluation of refractive outcomes, binocular distance and near visual acuity, enhancement procedures, spectacle independence, postoperative visual symptoms and patient satisfaction under routine clinical conditions. Another important strength is that all patients, including those who underwent enhancement procedures, remained within the study cohort and completed a minimum follow-up of 15 months after their last surgical intervention. Consequently, the reported visual and refractive outcomes represent the ultimate long-term clinical status following completion of the entire therapeutic pathway rather than outcomes after the primary procedure alone. This approach provides a realistic assessment of long-term clinical effectiveness from the patient’s perspective.

The present study has several limitations. First, its retrospective single-center design may limit the generalizability of the findings. Second, although all patients who underwent enhancement remained in the study cohort and were evaluated at least 15 months after their final surgical intervention, separate refractive and visual acuity measurements immediately before and after enhancement were not available because the study protocol included only predefined follow-up visits. Consequently, the isolated effect of enhancement could not be evaluated independently, and the reported outcomes represent the final long-term status after completion of treatment.

The emmetropic and anisometropic subgroups consisted of only three and two patients, respectively. Owing to these extremely limited sample sizes, no meaningful statistical inference could be performed, and the findings for these refractive categories are presented descriptively only. Therefore, no clinical conclusions should be drawn regarding the effectiveness or safety of Presbyond in emmetropic or anisometropic patients and larger studies are required to evaluate outcomes in these populations.

Although refractive analyses were performed at the eye level, inter-eye correlation was addressed using generalized estimating equations (GEE) with patient-level clustering, providing a more robust statistical approach than analyses assuming independence between fellow eyes.

The age-stratified analyses should be interpreted with particular caution. Several age categories contained only a small number of patients, while some categories were not represented, resulting in substantial variability and wide standard deviations. Consequently, these analyses should be regarded as exploratory and hypothesis-generating rather than confirmatory, and larger prospective studies with more balanced age distributions are needed to clarify the influence of age on long-term Presbyond outcomes.

Furthermore, patient-reported outcomes were not assessed using validated standardized questionnaires, which may limit comparability with studies employing formal quality-of-vision instruments.

## 5. Conclusions

Presbyond LBV provides durable long-term visual and refractive outcomes, excellent binocular distance and near visual acuity, and a high degree of spectacle independence in appropriately selected presbyopic patients. The favorable outcomes observed after surgery were maintained throughout extended follow-up after completion of all clinically indicated interventions, including enhancement procedures when required.

Nevertheless, long-term outcomes differed according to baseline refractive status. Compared with myopic patients, hyperopic individuals demonstrated higher enhancement rates, more frequent postoperative visual discomfort and lower subjective satisfaction despite generally favorable objective visual outcomes. These findings underscore the importance of careful patient selection, thorough preoperative counselling and realistic expectation management, particularly in hyperopic patients.

Future prospective multicenter controlled studies with larger cohorts, longer follow-up and standardized patient-reported outcome measures are warranted to optimize patient selection and identify predictors of the need for enhancement, postoperative visual symptoms, and long-term patient satisfaction. In addition, direct comparisons with other contemporary presbyopia-correcting strategies, including corneal- and lens-based approaches, are needed to better define the relative advantages, limitations, and appropriate indications of each treatment modality.

## Figures and Tables

**Table 1:** Baseline Characteristics by Refractive Group.

Group	Patients (*n*)	Age (Mean ± SD)	Age Range	Dominant Eye SE (Mean ± SD)	Range (D)	Non-Dominant Eye SE (Mean ± SD)	Range (D)
Myopic	53	47.603 ± 4.939	40–60	−4.371 ± 2.467	−10.63 to −0.50	−4.450 ± 2.423	−9.88 to −0.13
Hyperopic	42	52.167 ± 5.905	42–69	+2.056 ± 1.064	+0.50 to +4.88	+2.145 ± 1.321	+0.13 to +5.00
Anisometropic	2	47.50 ± 0.707	47–48	0.065 ± 0.092	0.00 to +0.13	−3.940 ± 1.146	−4.75 to −3.13
Emmetropic	3	49.667 ± 6.429	45–57	+0.127 ± 0.255	−0.13 to +0.38	0.123 ± 0.333	−0.13 to +0.50

**Table 2:** Refractive Outcomes (SE).

Refractive Group	Eye	S.E. Preoperative (Mean ± SD)	S.E. 1 Month (Mean ± SD)	S.E. ≥ 15 Months (Mean ± SD)	GEE *p*-Value (Preoperative vs. 1 Month)	GEE *p*-Value (1 Month vs. ≥15 Months)
Myopic (*n* = 53)	dominant	−4.371 ± 2.467	−0.308 ± 0.738	−0.472 ± 0.879	<0.001	0.003
	nondominant	−4.450 ± 2.423	−1.493 ± 0.729	−1.684 ± 0.866	<0.001	0.02
Hyperopic (*n* = 42)	dominant	2.056 ± 1.064	−0.259 ± 0.729	−0.053 ± 0.526	<0.001	0.051
	nondominant	2.145 ± 1.321	−1.543 ± 1.196	−1.367 ± 1.003	<0.001	0.178
Anisometropic (*n* = 2)	dominant	0.065 ± 0.092	−0.250 ± 0.000	0.060 ± 0.622	—	—
	nondominant	−3.940 ± 1.146	−1.380 ± 0.000	−1.440 ± 0.792	—	—
Emmetropic (*n* = 3)	dominant	0.127 ± 0.255	−0.167 ± 0.382	−0.293 ± 0.816	—	—
	nondominant	0.123 ± 0.333	−2.043 ± 0.437	−1.710 ± 0.709	—	—

“—” indicates descriptive analysis only because of insufficient sample size for reliable inferential testing.

**Table 3:** Binocular Visual Acuity Outcomes.

Refractive Group	Parameter	Preoperative Log MAR	1 Month log MAR	≥15 Months log MAR	*p*-Value (Preoperative vs. 1 Month)	*p*-Value (1 Month vs. ≥ 15 Months)	*p*-Value (Preoperative vs. ≥15 Months)
Myopic (*n* = 53)	DVA	0.002 ± 0.022	−0.001 ± 0.051	−0.017 ± 0.074	0.729	0.136	0.08
	NVA	0.300 ± 0.000	0.300 ± 0.000	0.300 ± 0.000	—	—	—
Hyperopic (*n* = 42)	DVA	0.002 ± 0.026	0.080 ± 0.119	0.017 ± 0.118	<0.001	0.002	0.405
	NVA	0.300 ± 0.000	0.293 ± 0.046	0.293 ± 0.046	0.323	1	0.323
Anisometropic (*n* = 2)	DVA	0.000 ± 0.000	0.000 ± 0.000	0.000 ± 0.000	—	—	—
	NVA	0.300 ± 0.000	0.300 ± 0.000	0.300 ± 0.000	—	—	—
Emmetropic (*n* = 3)	DVA	−0.027 ± 0.046	−0.027± 0.046	0.023 ± 0.093	—	—	—
	NVA	0.300 ± 0.000	0.300 ± 0.000	0.300 ± 0.000	—	—	—

“—” indicates descriptive analysis only because of insufficient sample size for reliable inferential testing.

**Table 4:** Efficacy and safety results.

Parameter	Myopic (*n* = 53)	Hyperopic (*n* = 42)	Overall (*n* = 100)
Final Binocular UDVA			
≤0.08 logMAR	47 (88.7%)	36 (85.7%)	87 (87.0%)
>0.08 logMAR	6 (11.3%)	6 (14.3%)	13 (13.0%)
Safety (CDVA line change)			
Loss ≥ 2 lines	6 (11.3%)	5 (11.9%)	12 (12.0%)
Loss 1 line	4 (7.5%)	3 (7.1%)	8 (8.0%)
No change	22 (41.5%)	24 (57.1%)	49 (49.0%)
Gain ≥ 1 line	21 (39.6%)	10 (23.8%)	31 (31.0%)
Reoperation rate	9 (17.0%)	12 (28.6%)	21 (21.0%)
Spectacle dependence			
Distance	5 (9.4%)	4 (9.5%)	9 (9.0%)
Near	4 (7.5%)	3 (7.1%)	8 (8.0%)
Intermediate	3 (5.7%)	1 (2.4%)	4 (4.0%)
Patient satisfaction	40 (75.5%)	27 (64.3%)	72 (72.0%)

**Table 5:** Age-stratified refractive stability expressed as the change in spherical equivalent (ΔSE) between the 1-month postoperative visit and the final follow-up (≥15 months). Statistical significance was evaluated using generalized estimating equations (GEE) accounting for inter-eye correlation.

Refractive Group	Age Group (Years)	*N* (Eyes)	Mean ΔS.E. ± SD (D)	*p*-Value
Myopic	40–45	40	−0.268 ± 0.359	<0.001
	46–50	38	−0.019 ± 0.549	0.872
	51–55	20	−0.162 ± 0.356	0.077
	56–60	8	−0.518 ± 1.001	0.07
	>60	0	—	—
Hyperopic	40–45	10	0.139 ± 0.706	0.549
	46–50	20	0.281 ± 0.571	0.032
	51–55	34	0.279 ± 0.511	0.015
	56–60	14	0.232 ± 0.787	0.29
	>60	6	−0.625 ± 1.883	0.453
Emmetropic	40–45	2	0.00 ± 0.00	—
	46–50	2	−0.065 ± 0.799	—
	51–55	0	—	—
	56–60	2	0.375 ± 0.177	—
	>60	0	—	—
Anisometropic	40–45	0	—	—
	46–50	4	0.125 ± 0.619	—
	51–55	0	—	—
	56–60	0	—	—
	>60	0	—	—

“—” indicates that inferential statistical analysis was not performed because of insufficient sample size.

**Table 6:** Age-stratified comparison of patient satisfaction, reoperation rates and postoperative spectacle dependence following Presbyond laser blended vision across refractive groups.

Refractive Group	Age Group (Years)	*N* (Patients)	Satisfaction *n* (%)	Enhancement *n* (%)	Postoperative Spectacle Dependence *n* (%)
Myopic	40–45	20	15 (75.0%)	3 (15.0%)	1 (5.0%)
	46–50	19	16 (84.2%)	2 (10.5%)	4 (21.1%)
	51–55	10	7 (70.0%)	2 (20.0%)	4 (40.0%)
	56–60	4	2 (50.0%)	2 (50.0%)	2 (50.0%)
Hyperopic	40–45	5	2 (40.0%)	3 (60.0%)	1 (20.0%)
	46–50	10	7 (70.0%)	2 (20.0%)	1 (10.0%)
	51–55	17	14 (82.4%)	1 (5.9%)	2 (11.8%)
	56–60	7	4 (57.1%)	3 (42.9%)	0 (0.0%)
	>60	3	0 (0.0%)	3 (100.0%)	2 (66.7%)

## Data Availability

Dataset available on request from the authors.

## Supplementary Materials

The following supporting information can be downloaded at: https://www.mdpi.com/article/10.3390/jcm15155850/s1, Supplementary File S1: Statystical analysis, Supplementary File S2: Presbyond GEE-Corrected Analysis.
